# Primary Ewing’s sarcoma of the uterine cervix: a case report and review of the literature

**DOI:** 10.1007/s00432-024-05698-2

**Published:** 2024-05-21

**Authors:** Yuhang Xiao, Yong Zhi, Guangxu Cao, Heling Ma, Jinli Gao, Fang Li

**Affiliations:** 1 Department of Gynecology, School of Medicine, Shanghai East Hospital, Tongji University, Shanghai, China; 2 Department of Pathology, School of Medicine, Shanghai East Hospital, Tongji University, Shanghai, China

**Keywords:** Cervix, Extraosseous Ewing’s sarcoma, Diagnosis, Chemotherapy, EWSR1–FLI1 gene fusion

## Abstract

**Background:**

Ewing’s sarcoma (ES) is an aggressive cancer of bone and soft tissue, most of which tend to occur in the bone. Extraosseous Ewing’s sarcoma (EES) of the cervix is extremely rare.

**Case presentation:**

In the present work, we reported a 39-year-old cervical EES patient with a 2.5*2.1*1.8 cm tumor mass. According to previous literatures, our case is the smallest tumor found in primary cervical ES ever. The patient initially came to our hospital due to vaginal bleeding, and then the gynecological examination found a neoplasm between the cervical canal and partially in the external cervical orifice. The diagnosis of EES was confirmed below: Hematoxylin & Eosin staining (H&E) revealed small round blue malignant cells in biopsy specimens. Immunohistochemistry (IHC) showed the positive staining for CD99, NKX2.2, and FLI1. Disruption of EWSR1 gene was found by fluorescence in situ hybridization (FISH), and the EWSR1–FLI1 gene fusion was determined by next-generation sequencing (NGS). The patient received laparoscopic wide hysterectomy, bilateral adnexectomy, pelvic lymphadenectomy, and postoperative adjuvant chemotherapy and remained disease free with regular follow-up for 1 year.

**Conclusions:**

Through a systematic review of previously reported cervical ES and this case, we highlighted the importance of FISH and NGS for the accuracy of ESS diagnosis, which could assist on the optimal treatment strategy. However, due to the rarity of the disease, there is no standard treatment schemes. Investigation on molecular pathological diagnosis and standardization of treatment regimens for cervical ES are critical to patients’ prognosis.

## Introduction

Ewing’s sarcoma (ES) is a malignant small round cell tumor that occurs overwhelmingly in the pelvis, ribs, and proximal long bones, with only about 20% occurring outside the bone (Riggi et al. 2021). As a rare and aggressive malignant tumor, based on morphological and immunophenotypic characteristics and the presence of chromosomal translocations, EES is part of the Ewing’s sarcoma family of tumors (ESFT) along with Ewing’s sarcoma of bone, primitive neuroectodermal tumor (PNET) and Askin’s tumor (Ewing’s sarcoma of the chest wall) (Grünewald et al. 2018). Primary EES has been reported to occur at various anatomical sites with less frequency: ileum (Li et al. 2017), breast (Papi et al. 2022), thyroid (Seipel et al. 2022), pancreas (Patel et al. 2020), and vagina (Tintila et al. 2021), but cervical EES is extremely rare.

Histopathology of EES has commonly featured a monotonous population of small round cells arranged in sheets or solid aggregates, separated by fibrous septa (Cidre-Aranaz et al. 2022). Pathological features were combined with immunohistochemistry (IHC) analysis, reverse transcription-polymerase chain reaction (RT-PCR) and fluorescence in situ hybridization (FISH) to confirm genetic translocations (Abboud et al. 2021). Notably, the identification of the fusion gene EWS/FLI (FLI, a member of the ETS family of transcription factors) caused by the characteristic chromosomal translocation t(11;22)(q24;q12) is the molecular diagnostic feature of ESFT, occurs in 85% of individuals with ES (Balamuth and Womer 2010). In the remaining approximately 15% of cases, chromosomal translocations can also result in other genes fusions between the EWS and ETS family, such as ERG, ETV1 or E1AF (Riggi and Stamenkovic 2007). Diagnostic imaging aspects contain magnetic resonance imaging (MRI) and fluorodeoxyglucose-positron emission tomography (FDG‑PET) are used for local staging and exclusion of metastasis (Meyer et al. 2008). In terms of treatment, EES is multimodal therapy including of induction chemotherapy and local therapy (surgery and/or radiotherapy) (Cidre-Aranaz et al. 2022). Given the lack of effective clinical trials and valuable analyses due to the rarity of EES, local treatment and radiotherapy strategies mostly follow the guidelines of classical bone Ewing’s sarcoma (Gerrand et al. 2020). To date, there is no consensus on the efficient diagnosis and optimum treatment for cervical ES.

Here, we described an exceedingly rare case of primary cervical ES and reviewed previously published reports including clinical features, diagnosis, and treatment from limited clinical literature. We further emphasized the significance of elaborative pathologic diagnosis and neoadjuvant chemotherapy in cervical ES.

## Case presentation

A 39-year-old woman presented to our hospital with prolonged menstrual periods doubled menstrual flow and a small amount of intermenstrual bleeding. A neoplasm in the external os of the cervix with a wide root was found through the gynecological examination. During the hysteroscopy, a 2.5*2.1*1.8 cm brittle and fragile neoplasm was found to be located in the posterior wall of the cervical canal and was removed for pathological examination. Postoperative pathological examination and H&E staining showed that there were small round blue tumor cells. Immunohistochemical results showed positive expression of CD99, NKX2.2 and FLI1. The pathological features were highly consistent with the diagnosis of ES. To further confirm the diagnosis, FISH examination revealed a break in the EWSR1 gene. The results of next-generation sequencing of tumor tissue showed that the EWSR1 and FLI1 genes were fused to form a new gene with transcriptional function. The patient was diagnosed with EES of the cervix based on the above examination results. Whole-body PET-MRI showed a hypermetabolic uterine cervical lesion without any indications of metastatic sites.

The surgical options were laparoscopic wide hysterectomy, bilateral adnexectomy, and pelvic lymphadenectomy. The operation went smoothly, with a bleeding volume of about 200 ml, and the patient was in good health after the operation. Postoperative gross specimens showed no residual malignant tumor with negative margin, and no lymph node metastasis. The clinical stage of the disease was IB2 according to the FIGO staging system. The patient was treated with six cycles of postoperative adjuvant chemotherapy, consisting of vincristine, doxorubicin, and cyclophosphamide alternating with ifosfamide and etoposide (VDC/IE). The patient remains free of metastases 1 year after initial diagnosis and has a good functional prognosis.

## Diagnostic assessment

**Fig. 1:**
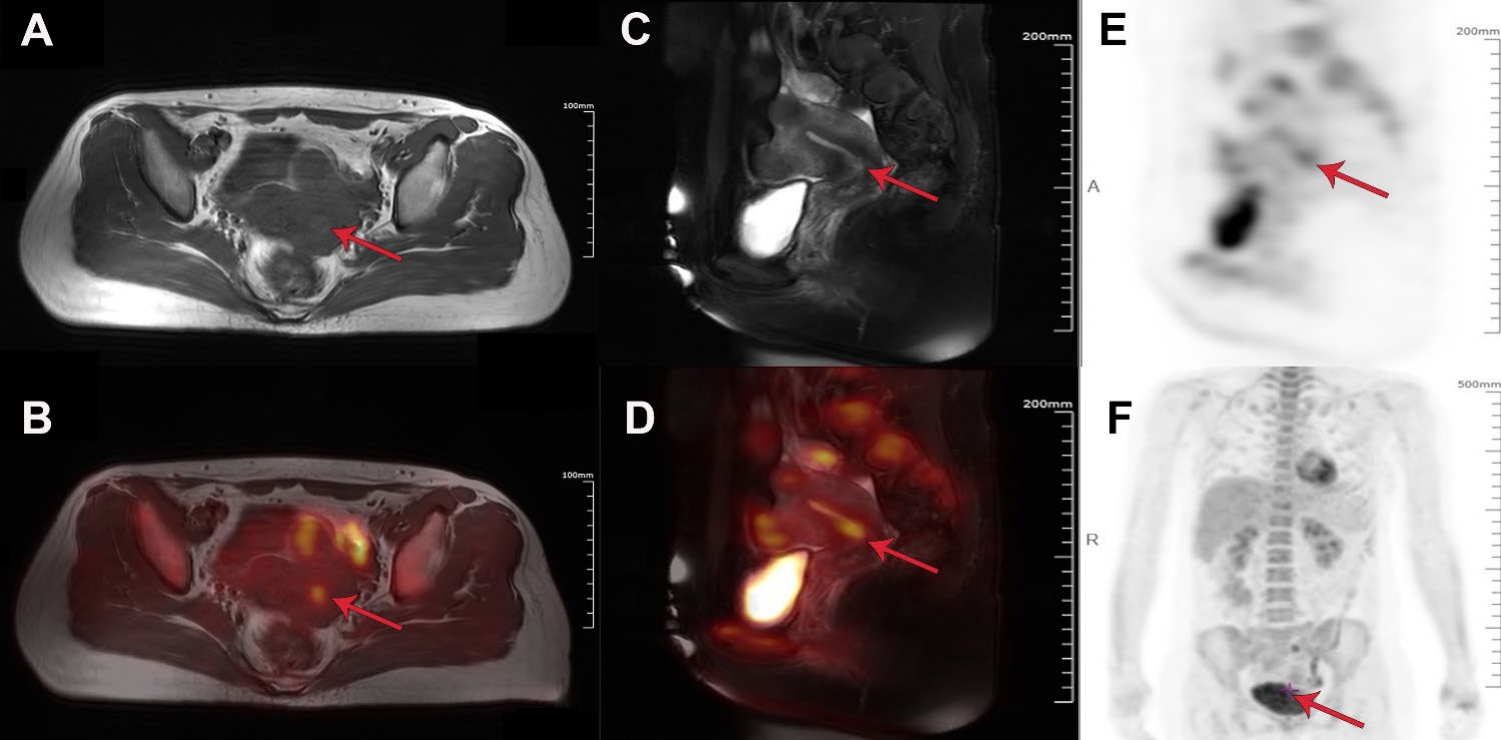
Positron emission tomography-magnetic resonance imaging (PET-MRI) of the ES patient. **A**–**B** An axial T2-weighted MRI image and an axial FDG PET-MRI fused image. **C**–**D** A coronal T2-weighted MRI image and a coronal FDG PET-MRI fused image. **E**–**F** Coronal FDG PET images performed simultaneously with MRI anterior and right. Increased FDG metabolism in the anterior wall of the cervical segment of the lower uterine body (red arrows)

T2-weight spin echo imaging without fat suppression in the transaxial plane revealed a heterogeneous high-intensitive mass in the uterine cervix and multiple echoes with heterogeneous high intensity in the uterine myometrium (Fig. 1A). Fused images of FDG pet and T2-weighted spin echo imaging without fat suppression revealed slightly increased FDG metabolism in the anterior wall of the cervical segment of the lower uterine body. Images show multiple uterine fibroids with abnormally increased FDG metabolic uptake and degeneration of anterior wall fibroids (Fig. 1B). Furthermore, coronal slices of PET/MRI also showed multiple elevated FDG metabolism in the uterus and internal cervical os, which further suggested that the presence of multiple uterine fibroids and cervical malignancies (Fig. 1C–F).

**Fig. 2:**
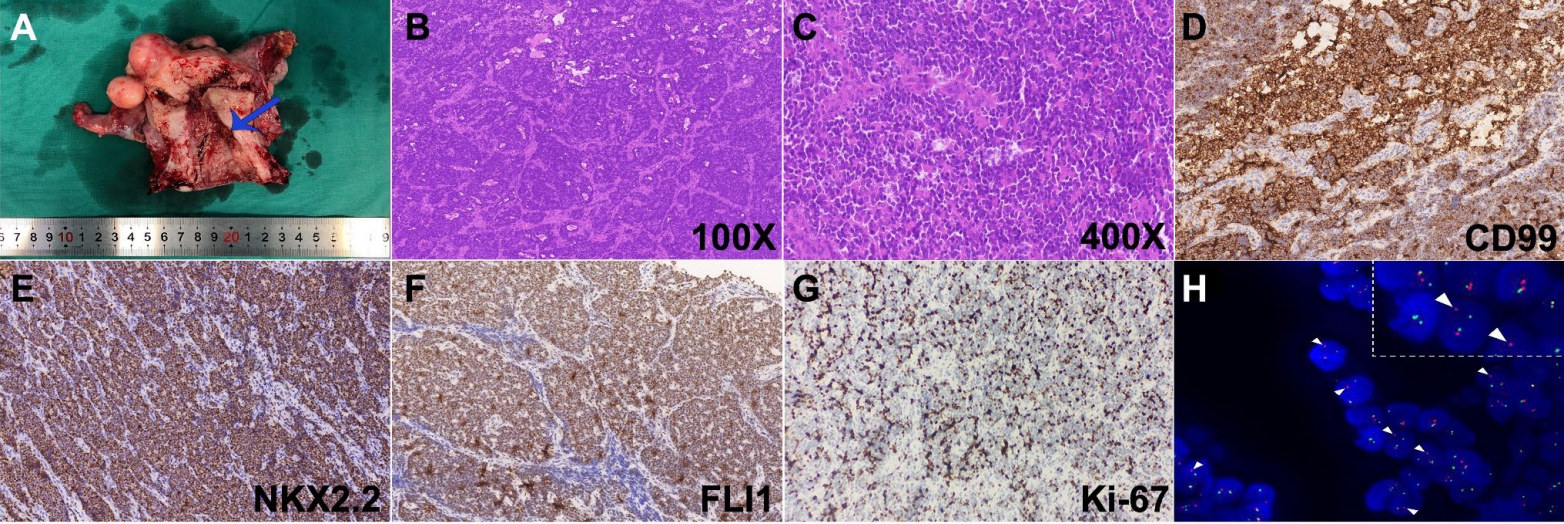
Characteristics of the ESS tumor were detected with FISH through a two-color (red/green) tomographic probe. The H&E staining and immunohistochemistry (IHC) of the cervical ES. **A** The blue arrow indicates the presence of residual ES on the resected uterus. **B**–**C** H&E staining identified the morphological cellular malignant tumor composed of small, blue, round, and pleomorphic cells with vesicular nuclei under 100× and 400× magnification. **D**–**G** The IHC of tumor tissue is stained for CD99, NKX2.2, Ki-67, and Fli-1. All images were taken under 200X magnification. **H** A split signal pattern (arrowheads) signifying disruption of EWSR1 was showed through break-apart FISH

Gross pathological specimens of the uterus after wide hysterectomy demonstrated no obvious tumor tissue (Fig. 2A). Histopathological analysis revealed that the morphology of tumor cells exhibiting a small round shape with oval or round nuclei, sparse eosinophilic cytoplasm, uniform distribution of chromatin, and few mitoses (Fig. 2B–C). IHC analysis showed the positive rate of Ki-67 between 50 and 70%. In addition, malignant tumor markers including CD99, NKX2.2, and FLI1 were all highly expressed in tumor cells (Fig. 2D–G). The diagnosis of ES was further confirmed by the characteristic markers CD99( +) and NKX2.2( +). Meanwhile, the IHC results showed that the following markers were not expressed: CK( – ), CD10( – ), EMA( – ), MyoD1( – ), Desmin( – ), and Myogenin ( – ). Overall, the results above could exclude epithelial tissue and provide further support for the diagnosis of EES. The tumor was detected by FISH with the 5′ and 3′ ends of the EWS gene were labeled with dual color (red/green) break-apart probe. The results showed that the green and red signal separation of 52% of the cells (per 100 cells), which proved that EWS gene disruption occurred in this case (Fig. 2H).

**Fig. 3:**
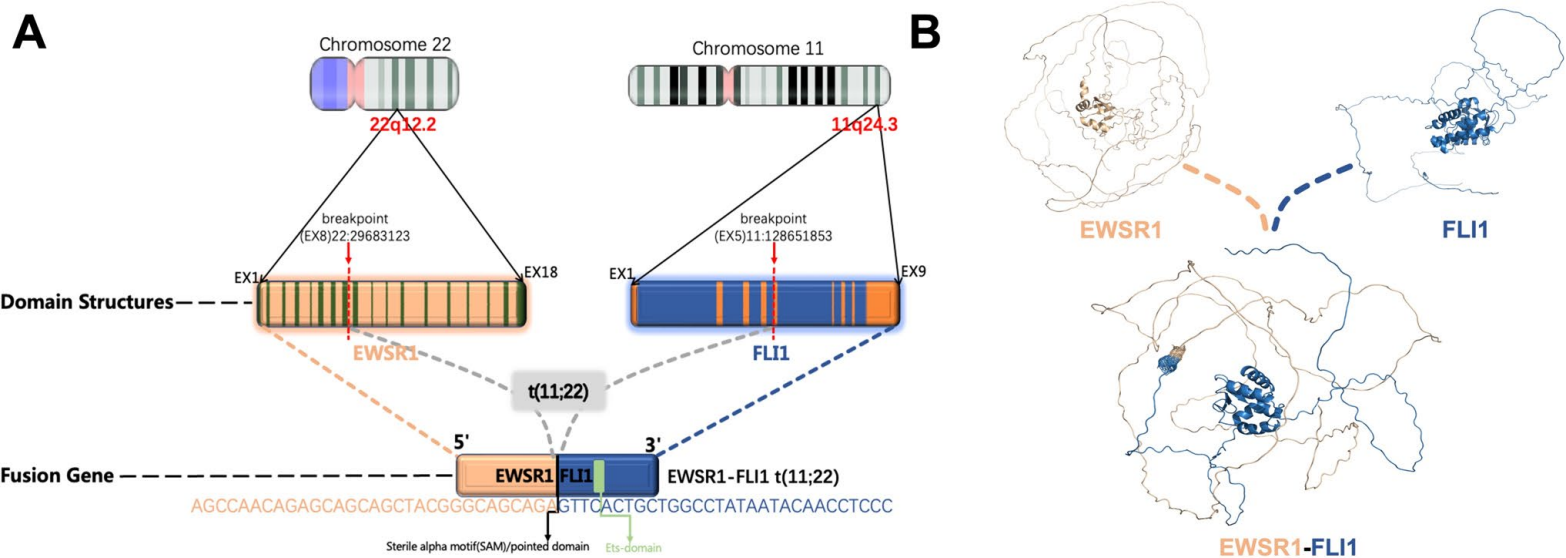
The schematic diagram of the fusion gene EWSR1/FLI1 revealed by NGS. **A** The presentation of genetic rearrangement analysis. (Gene: EWSR1–FLI1; Fusion gene transcripts: NM_013986.3/NM_002017.4; Exon: EX8:EX5). **B** Prediction of the tertiary structure of the protein encoded by the EWSR1–FLI1 fusion gene by PyMOL software. The wheat line is the part encoded by the EWSR1 and the blue line is the part encoded by FLI1. The mesh structure is the fusion site of EWSR1 and FLI1 at the predicted tertiary structure level of the protein

ES is characterized by reciprocal chromosomal translocations, resulting in chimeric fusion proteins that influence tumorigenesis and progression (Grünewald et al. 2018). Up to 85% of ES cases can be detected by chromosomal translocation t (11; 22) caused by EWSR1–FLI1 gene fusion. The N-terminal region of EWSR1gene (Ewing sarcoma breakpoint region 1 gene) binds to the C-terminal region of FLI1 (Friend leukemia integration 1) (Delattre et al. 1992), a transcription factor containing the DNA-binding domain. EWSR1–FLI1 is involved in major cell-cycle regulation, proliferation and response to DNA damage in ES (Kauer et al. 2009). Interestingly, the next-generation sequencing (NGS) results showed that the EWSR1 and FLI1 gene were fused, and consequently construct a novel fusion gene EWSR1–FLI1. The fusion site was in exon 8 of EWSR1 gene and exon 5 of FLI1 gene. The 5′ end of the EWSR1–FLI1 fusion gene retains the EWSR1 gene promoter to exon 8, and the 3′ end retains the FLI1 gene exon 5 to the stop codon (Fig. 3A). The EWSR1–FLI1 fusion gene acts as an aberrant transcription factor, where EWSR1 provides the transcriptional activation domain and FLI1 provides the DNA-binding structural domain. EWSR1–FLI1 is the main determinants of the regulatory activity of a great deal of enhancers shared by Ewing’s cell lines and primary ES (Riggi et al. 2014). EWS–FLI1 promotes ES survival and differentiation regulatory programs through direct induction or inhibition of enhancers. Next, we modeled the tertiary structure of the amino acid sequence encoded by the EWSR1–FLI1 fusion gene using PyMOL software (Rigsby and Parker 2016) (Fig. 3B). This protein is closely related to both DNA-binding transcription factor activity and regulation of transcription by RNA polymerase II.

## Discussion

The most prevalent site of ES is bone, and occurrence in the cervix is extremely rare. In this work, we reported a 39-year-old cervical ES and summarized all reported cases of cervical ES (Table 1). In total 28 cervical ES cases, ranging in age from 13 to 59 years old with a median of 35.5 years. Most patients experience abnormal vaginal bleeding and lower abdominal pain. ES had a defined cervical growth with a minimum diameter of 3 cm and a maximum diameter of 10 cm. Our cervical EES patient had a tumor size of 2.5*2.1*1.8 cm, and it is the smallest tumor found in primary cervical ES ever. Clinical staging data were missing for 7 patients. The FIGO clinical stage in 9 patients was IV (43%), indicating that the high rate of metastasis of this disease deserves our attention. There were nine patients with stage IB1 or IB2 (43%), two patients with stage II (10%), and one patient with stage III (5%). In our review, the vast majority of cervical ES patients (79%) underwent surgical treatment. All surgical patients included extensive total hysterectomy, and 11 patients (50%) of which were extensive total hysterectomy (ETH), bilateral salpingo-oophorectomy (BSO), and pelvic lymphadenectomy (PL). Except one case without reliable evidence, 24 patients (86%) received postoperative chemotherapy (the smallest tumor size was 3 cm), but the chemotherapy regimen was heterogeneous. Of the 12 known patients with a defined chemotherapy regimen, 8 cases (66%) cases had VAC included in their chemotherapy regimen (tumor size ranged from 5.0*4.0 cm to 9.8*8.5*7.8 cm, stage IV), with VAC alone in 3 (25%) and VAC + IE in the remaining 5 (3; One patient underwent 2 cycles of etoposide and cisplatin after VAC 1 cycle; VAC; was added after VIDE in 1 case). Follow-up ranged from 1 month to more than 10 years. 11 patients eventually died, 8 of them within 1 year. Notably, all patients who died within 6 months had tumor size > 7 cm or had systemic metastases. Therefore, tumor size and metastasis at the time of diagnosis can be important prognostic factors.

**Table 1:** Summary of previous reported cervical ES with clinical features, treatment options, and outcome

Age	Size (cm)	FIGO stage	Surgery	Chemotherapy regimens	RT	Outcome	References
13	5.0 × 4.5 × 4.0	IVB	ETH + BSO + PL	YES	+	D,8 months	Cheng et al. (2021)
17	4.0	NE	ETH + PL	YES	–	A, > 10 years	Ward et al. (2000)
19	NE	NE	NE	VAC after RT	+	A, on treatment when reported	Goda et al. (2006)
21	5.5	IB2	ETH	DIME, 6 cycles; VIA,5 cycles	–	A,27 months	Snijders-Keilholz et al. (2005)
23	10.5 × 7.0 × 6.0	IV	ETH + BSO	Adjuvant Cisplatin 1 cycle	–	D,12 days	Masoura et al. (2012)
23	9.8 × 8.5 × 7.8	NE	ETH + BSO + PL	etoposide–cisplatin 2 cycles after VAC 1 cycle	+	A,4 years	Arora et al. (2012)
24	7.0 × 4.0 × 4.0	IVB	ETH + BSO	YES	–	D,3 months	Cheng et al. (2021)
24	8.9 × 6.8 × 7.2	NE	ETH + BSO + PL	VAC, 2 cycles	+	A,2 years	Tsao et al. (2001)
26	10.0 × 9.0 × 6.0	IVB	ETH	YES	+	D,36 months	Cheng et al. (2021)
26	NE	IB1	ETH + BSO + PL	Cisplatin and 5FU	+	D,50 months	Horn et al. (1997)
27	5.5	IIIB	NO	VAC and IE	+	A,6 months	Li et al. (2013)
28	6.0	IB2	ETH + BSO + PL	VAC	–	A,33 months	Khosla et al. (2014)
29	3.5 × 2.8 × 2.0	NE	ETH + BSO	YES	+	D,51 months	Cheng et al. (2021)
35	4.0	IB1	ETH + BSO + PL	YES	–	A,18 months	Malpica and Moran (2002)
36	7.0	IB2	ETH	NO	–	A,18 months	Cenacchi et al. (1998)
37	4.5 × 4.5 × 3.5	NE	ETH + BSO + PL	YES	–	NE	Cheng et al. (2021)
40	5.4	IVB	NO	YES	+	D,11 months	Cheng et al. (2021)
42	4.0 × 3.9 × 3.4	NE	ETH + BSO	YES	+	A,57 months	Cheng et al. (2021)
44	7.0 × 6.5 × 5.0	IB2	ETH + PL, left SO	YES	–	A,6 months	Sato et al. 1996)
45	5.0 × 4.0	IB2	ETH + BSO + PL	VAC and IE	–	A,4 years	Farzaneh et al. (2011)
45	8.0	IB2	ETH	NO	+	A,42 months	Pauwels et al. (2000)
45	7.1 × 6.3 × 5.7	IVB	NO	NO	–	D,3 months	Jia et al. (2022)
47	9.8 × 8.9	IVB	NO	YES	+	A,18 months	Cheng et al. (2021)
49	5.3*4.8*6.6	IIB	ETH + BSO	Adjuvant VAC and IE	+	D,10 months	Mashriqi et al. (2015)
51	3.0	IB2	ETH + BSO + PL	YES	–	A,18 months	Malpica and Moran (2002)
52	NE	IIA	ETH + BSO + PL	PVB, 2 cycles	NE	D,9 months	Xiao et al. (2014)
57	NE	IVB	NO	VAC after VIDE	+	A,18 months	Bílek et al. (2015)
59	NE	IVB	ETH + BSO + PL	NE	NE	D,15 days	Xiao et al. (2014)

*ETH* extensive total hysterectomy, *BSO* bilateral salpingo-oophorectomy, *PL* pelvic lymphadenectomy, *NE* no evidence, *VAC* Vincristine–Adriamycin–Cyclophosphamide, *DIME* Doxorubicin, Ifosfamide–Mesna–Etoposide, *VIA* Vincristine–Ifosfamide–Dactinomycin, *5FU* 5-fluorouracil, *IE* Ifosfamide–Etoposide, *PVB* Cisplatin–Vincristine–Bleomycin, *VIDE* Vincristine–Ifosfamide–Doxorubicin–Etoposide, *RT* radiation therapy, *A* alive, *D* died

Imagological examination plays an important role in the diagnosis and staging of EES (Javery et al. 2011). There is a challenge in diagnosing EES due to less sensitivity of ultrasonography and computed tomography (CT) (Papi et al. 2022). PET may be more efficient for diagnosis, staging and monitoring response to therapy due to ES/PNET tumors are sensitive to fludeoxyglucose (Mashriqi et al. 2015). However, the definitive diagnosis of EES is more dependent on the histopathological analysis. Histopathology revealed that tumors consist of a monotonous population of small round cells which are arranged in solid clumps or sheets, separated by fibrous septa. There are sometimes focal rosettes present in the cells. Tumor cells have round nuclei, coarse chromatin, small nucleoli, and scant to abundant eosinophilic or clear cytoplasm. It is noted that mitotic activity is brisk, and that necrosis is prevalent in the cells (Gibbs et al. 2016). Immunohistochemically, the overwhelming majority of EES is diffuse, strong, membranous CD99 expression which was encoded by MIC2 gene (Abboud et al. 2021; Fouchet et al. 2000). CD99 is a sensitive diagnostic marker for EES but is not specific because it is also seen in other tumors such as rhabdomyosarcoma (Bernstein et al. 2006). Other nonspecific markers that have been reported include broad-spectrum cytokeratins, desmin, myogenin, synaptophysin, chromogranin, CD117, and CD56 (Chougule et al. 2019; Pohar-Marinsek 2008; Oliveira et al. 2013).

The most characteristic chromosomal translocation to ES is t(11;22) (q24;q12) (Maroun et al. 2019; Abboud et al. 2021). The definitive chromosomal translocation of EES is the fusion of Ewing’s Sarcoma Breakpoint Region 1(EWSR1) gene with the DNA fusion domain of the ETS transcription factor gene (Embree et al. 2009). The FLI1 transcription factor belongs to the ETS family of transcription factors. The EWSR1–FLI1 fusion gene (85%–90% of ES cases) is the most common type, and the rest of the gene fusion with EWSR1–ERG (10%), EWSR1–ETV1 (< 1%), EWSR1–E1AF (< 1%), EWSR1–FEV (< 1%), EWSR1–ERG (< 1%) (Riggi et al. 2021). EWSR1–FLI encodes a chimeric transcription factor that act as a strong transactivator of promoters containing FLI1 (Bailly et al. 1994). Upregulation of EWSR1–FLI1 expression is closely associated with ES development, induction of cell survival, metastatic spread, and acquisition of self-renewal properties (Kim et al. 2022). In this work, we found EWSR1–FLI1 gene fusion by NGS is crucial for confirmation the definitive diagnosis.

Due to the rarity of ES occurrence, both the National Comprehensive Cancer Network (NCCN) (Biermann 2013) and the European Society of Medical Oncology (ESMO) (Casali et al. 2018) have recommended that EES should follow the same treatment regimen as other members of the ES family. Additionally, Weshi et al. also showed that adult EES were the same as those of bone Ewing’s sarcoma in terms of response to multimodal therapy and prognostic factors affecting the treatment outcome (El Weshi et al. 2010). Although the ultimate therapeutic benefit of EES depends on appropriate surgical resection, aggressive chemotherapy, and local adjuvant radiotherapy, the current optimal management and course of ES occurring in the cervical site are unknown. Given ES in the cervix is rare, there are no guidelines to guide clinicians to clear treatment options. We reported that cervical ES in the aspect of operation method, postoperative adjuvant radiotherapy and chemotherapy to guide clinicians to choose the treatment in the future by combining our latest cases.

Previous studies have demonstrated that local treatment (Surgery and/or radiotherapy) can significantly improve the survival rate of EES (Rud et al. 1989). Initial tumor size, as an important prognostic tool for this disease, together with the appearance of metastases and the margin of surgical resection, affects overall survival (OS) and event-free survival (EFS) (Foulon et al. 2016). Chemotherapy as conventional medication that improve the OS rate and reduce the possibility of recurrence in patients (Boussios et al. 2018). A multiagent chemotherapy including VDC/IE is commonly regarded as the first-line therapy be effective for ES in large combination trials (Pappo and Dirksen 2018). Among the 28 cases of cervical ES reviewed, only 3 (11%) cases did not receive chemotherapy, and one of them developed a vertebral metastasis and paraplegia prior to chemotherapy (Jia et al. 2022). In general, the OS and PFS of EES patients received VDC/IE regimen and standard local control modalities were comparable to classic bone ES. Unfortunately, EES patients still had a significantly higher risk of local failure (Salah et al. 2020). Adjuvant chemotherapy should be given in all cases of EES regardless of marginal status. This emphasized the rarity of cervical ES and the urgent need for clear clinical chemotherapy regimens and plans.

In conclusion, we described an especially rare case of a 39-year-old primary cervical ES. The tumor size is 2.5*2.1*1.8 cm, which is the smallest diameter cervical ES reported according to our knowledge. Due to the rarity of this disease, we emphasized early pathological diagnosis and multidisciplinary consultation in the differential diagnosis of cervical tumors to detect this rare disease and determined the optimal treatment strategy. Through a systematic review of previously reported cervical tumors, we found that the major of these cases underwent wide excision, with chemotherapy regimens consistent with classical bone ES, combined with radiotherapy when necessary. Future management principles of prospective for cervical ES should be investigated.

## Data Availability

The relevant data and materials pertaining to this study are available upon request.
